# Receipt of seasonal malaria chemoprevention by age-ineligible children and associated factors in nine implementation states in Nigeria

**DOI:** 10.1186/s12936-024-04916-z

**Published:** 2024-03-30

**Authors:** Taiwo Ibinaiye, Kunle Rotimi, Ayodeji Balogun, Adaeze Aidenagbon, Chibuzo Oguoma, Christian Rassi, Kevin Baker, Olusola Oresanya, Chuks Nnaji

**Affiliations:** 1 Malaria Consortium, 33 Pope John Paul Street, Maitama, Abuja, FCT Nigeria; 2 Malaria Consortium, No.24 Randa Area, Ogbomoso, Nigeria; 3 Malaria Consortium, The Green House, 244-254 Cambridge Heath Road, London, E2 9DA UK; 4 Department of Global Public Health, Karolinska Institute, Stockholm, Sweden

**Keywords:** Malaria, Seasonal malaria chemoprevention, Coverage, Eligibility, Programme improvement

## Abstract

**Background:**

As part of implementation quality standards, community distributors are expected to ensure that only age-eligible children (aged 3–59 months) receive seasonal malaria chemoprevention (SMC) medicines during monthly campaigns. There is uncertainty about the extent to which SMC medicines are administered to ineligible children. This study aimed to assess the magnitude of this occurrence, while exploring the factors associated with it across nine states where SMC was delivered in Nigeria during the 2022 round.

**Methods:**

This analysis was based on data from representative end-of-round SMC household surveys conducted in nine SMC-implementing states in Nigeria. Data of 3299 age-ineligible children aged > 5 years and their caregivers were extracted from the survey dataset. Prevalence of receipt of SMC medicines by ineligible children was described by child-, caregiver- and SMC-related factors. Mixed-effects multivariable logistic regression models were fitted to explore the factors associated with ineligible receipt of SMC medicines.

**Results:**

30.30% (95% CI 27.80–32.90) of ineligible children sampled received at least one dose of SMC medicines in 2022, the majority (60.60%) of whom were aged 5–6 years while the rest were aged 7–10 years. There were lower odds of an age-ineligible child receiving SMC medicines among caregivers who were knowledgeable of SMC age eligibility (OR: 0.53, 95% CI 0.37–0.77, p < 0.001), compared with those who were not knowledgeable of age eligibility. Higher odds of receipt of SMC were found among age-ineligible children whose caregivers had higher confidence in the protective effect of SMC against malaria (OR: 2.01, 95% CI 1.07–3.72, p = 0.030), compared with those whose caregivers were less confident. Compared with ineligible children of younger caregivers (aged < 20 years), those whose caregivers were older had lower odds of receiving SMC than those whose caregivers were younger; with lower odds among children of caregivers aged 20–39 years (OR: 0.50, 95% CI 0.30–0.82, p = 0.006).

**Conclusions:**

This study contributes important evidence on the magnitude of the receipt of SMC medicines by age-ineligible children, while identifying individual and contextual factors associated with it. The findings provide potentially useful insights that can help inform and guide context-specific SMC implementation quality improvement efforts.

## Background

Malaria is a significant public health concern in Nigeria, with the country accounting for 38.4% of global malaria deaths in children under 5 years [1]. To reduce the burden of malaria in this age group, Nigeria adopted seasonal malaria chemoprevention (SMC) as a malaria prevention strategy in 2014 [2, 3]. SMC typically involves the intermittent administration of a combination of two antimalarial medicines—sulfadoxine–pyrimethamine and amodiaquine (SPAQ)—to children aged 3–59 months during the peak malaria transmission season, typically coinciding with the rainy season [4]. This approach has demonstrated effectiveness in reducing malaria incidence, morbidity, and possibly mortality [4–6].

SMC is typically delivered door-to-door by community distributors, who are trained to determine the age of the child through birth certificate, immunization cards, or caregiver confirmation if those documents are not available. The first doses of SPAQ are administered through directly observed treatment (DOT) [7]. However, in low and middle-income countries, civil registration and identification systems are often underdeveloped [8–11]. Coupled with the high prevalence of malnutrition and stunting in regions with high malaria attack rates, determining children’s age with accuracy is, therefore, often challenging [6]. As a result, age-ineligible children, that is, those who are either too young or too old to receive SMC [12], may inadvertently receive SMC medicines. Moreover, because SMC is often regarded as a highly effective malaria prevention intervention within SMC target populations, community distributors may face pressure from caregivers to provide SPAQ to older children [13]. The administration of SMC medicines to age-ineligible children is an important indicator of the quality of SMC delivery and can be used to track and identify implementation quality and fidelity gaps [14]. Receipt of SMC medicines by ineligible children presents various challenges. The SPAQ tablets currently used in SMC are available in two age-based formulations: a lower-strength formulation for children 3–< 12 months and a higher-strength formulation for children 12–59 months. If administered correctly, these formulations provide a high degree of protection from malaria for approximately 28 days [15]. For older children, the above formulations are unlikely to offer sufficient anti-malarial drug concentrations in the blood to give protection for the entire 28-day cycle, and so are likely to contribute to the development of drug-resistant *Plasmodium falciparum* malaria [16]. Furthermore, the administration of medicines to children outside of the designated age range complicates the quantification of SPAQ needs and procurement, which can potentially lead to stock-outs and eligible children missing out on this life-saving intervention [16, 17]. While evidence from routine SMC coverage surveys suggest that the administration of SMC to ineligible is common, the extent to which this happens remains uncertain as currently available evidence is based on routine coverage surveys which are often not powered to provide representative results on the proportions of ineligible children receiving SMC medicines. Moreover, there is little evidence on the factors influencing the administration of SMC medicines to ineligible children.

This study, therefore, aims to assess the receipt of SMC medicines by age-ineligible children and associated factors in nine implementation states in Nigeria. The findings of this study will be useful in guiding the development of strategies to improve the quality delivery of SMC in Nigeria, strengthen the current evidence base, and provide additional datapoints for informing SMC programme improvements in similar implementation contexts.

## Methods

### Study design

The study is based on comprehensive end-of-round cross-sectional household surveys conducted in nine states where SMC was delivered in Nigeria in 2022. Survey methods are described in greater detail elsewhere [18].

### Study setting

**Fig. 1:**
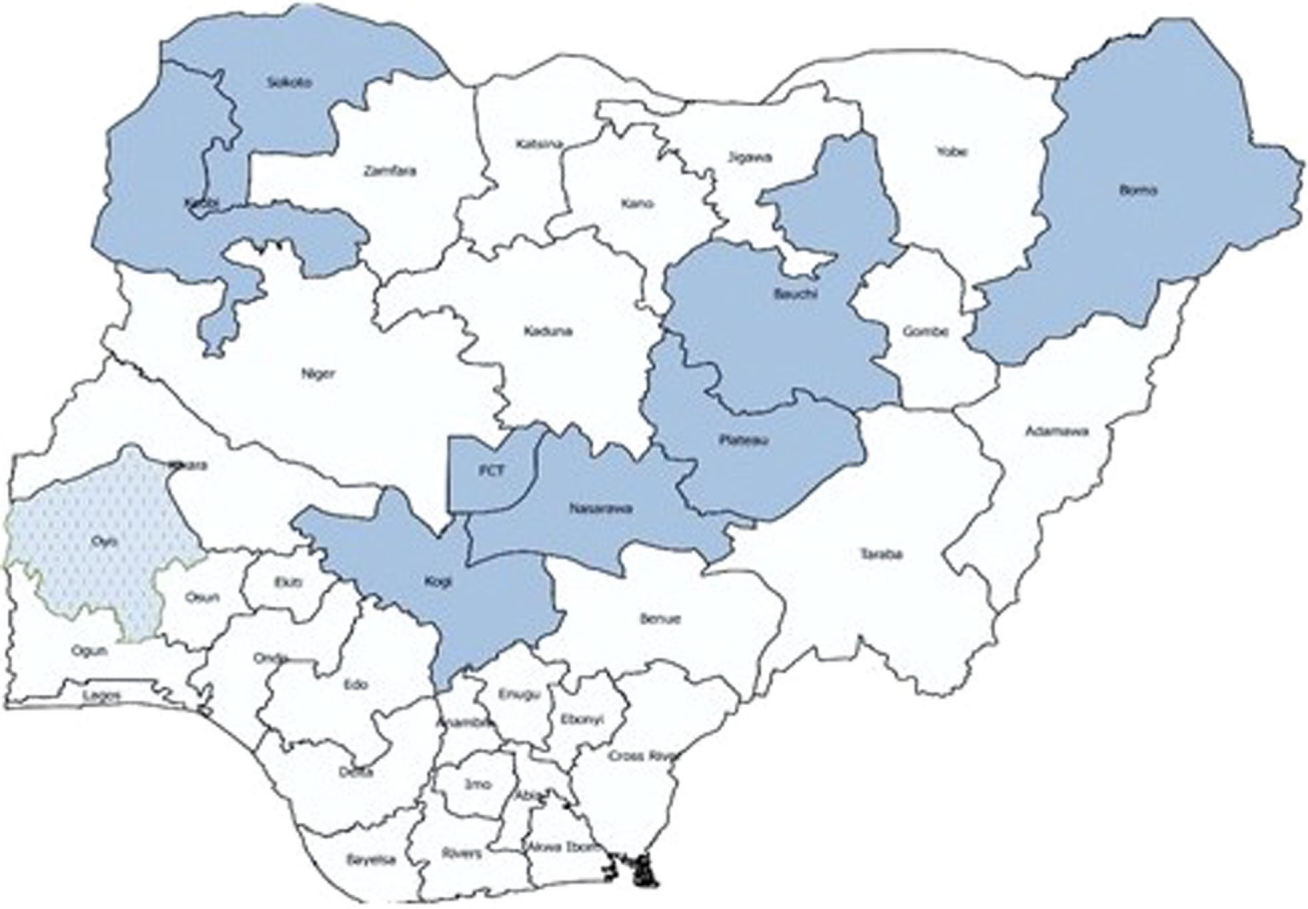
Map of Nigeria illustrating the states represented in this analysis

This study used data from SMC campaigns implemented in Bauchi, Borno, Kebbi, Kogi, Nasarawa, Oyo, Plateau, Sokoto States, and the Federal Capital Territory (FCT) Abuja in 2022 (see Fig. 1). SMC was introduced in the FCT, Oyo state and some Local Government Areas (LGAs) in Kogi state that year, whereas the other states and LGAs in Kogi state had previous experience of implementing SMC. Five monthly cycles were implemented in the FCT, Kogi, Nasarawa, Oyo, Plateau and 10 LGAs in Bauchi state, while four cycles were implemented in Borno, Kebbi, Sokoto and another 10 LGAs in Bauchi state. The five-cycle SMC round was implemented from early June to early October 2022 whereas the four-cycle round was delivered from late June to late September 2022. Around 10.72 million SMC eligible children aged 3–59 months were targeted across the eight states and the FCT in 2022.

### Sampling and data collection process

Surveys were carried out after the last monthly SMC cycle by independent evaluators. A multistage cluster sampling technique was used to select households with SMC-eligible children aged 3–59 months. Surveys were intended to achieve a representative sample of the target population of eligible children at country level and state levels. Sampling protocols aimed to achieve a self-weighted sample with sampling units selected with probability proportional to size. Only at the last stage of sampling (i.e. at the compound level) was a constant number of eligible children (one child per household) selected. Older children aged 5–10 years (the focus of this analysis), if present in sampled households, were randomly selected to estimate the degree to which ineligible children received SMC as a measure of implementation quality. The analytical sample of older children included in this analysis thus represents a subset of the entire survey sample across the nine participating states. The primary outcome was SMC coverage in age-ineligible children (proportion of age-ineligible children who received at least one dose of SMC medicines). Secondary outcomes included SMC adherence (receipt of the full 3-day course), awareness, knowledge and confidence (belief). Data were collected using structured questionnaires and analysed using descriptive and inferential statistics. Independent variables included child-level factors, including age and sex; caregivers’ sociodemographic characteristics, such as age, sex, level of education, occupation status and SMC-specific outcomes such as caregivers’ SMC awareness, knowledge, and confidence. The study required a minimum sample size of 1475 caregiver-child pairs to be powered to 80%, at the 95% confidence level using a two-tailed test and 5% margin of error, to estimate the prevalence of SMC coverage among age-ineligible children (aged 5–10 years). This assumes SMC coverage of 20% among age-ineligible children, based on evidence from routine programme data. Data were pooled across all nine participating states to achieve the estimated sample size.

### Statistical analysis

Descriptive statistics were used to summarize the data, by presenting the distributions of independent variables by the outcome variable. The distributions were expressed as frequencies and percentages for categorical variables and means and their standard deviations for continuous variables. Bivariate analyses were used to examine the crude association between each independent variable and receipt of SMC medicines by children aged 5–10 years. Mixed-effects multivariable logistic regression was used to identify predictors of receipt of SMC medicines by age-ineligible children by examining the adjusted association between the outcome variable and child- and caregiver-level covariates through mutual adjustment. Given the hierarchical nature of the data and to account for the clustering effect, the analysis followed a mixed-effects logistic regression approach in fitting the model, with random intercepts for cluster units (wards). Measures of association were presented as odds ratios (OR) with their corresponding 95% confidence intervals (CI), with statistical significance considered at p-value < 0.05. The Akaike information criterion (AIC) was used to assess the goodness-of-fit of the model. Variance Inflation Factor (VIF) was applied to test for multicollinearity. Data analysis was performed using Stata statistical software (Version 16).

### Ethical considerations

Ethical approval for the surveys was granted by the National Health Research Ethics Committee in Nigeria (NHREC Approval Number NHREC/01/01/2007-14/10/2022). Data were used in accordance with the NHREC’s ethics standards. Informed consent was obtained from all survey participants before data collection.

## Results

Figure 1 shows states where SMC was delivered in 2022 as included in this analysis.

The analytic sample included data from a total 3299 caregivers of age-ineligible children aged 5–10 years across the nine states without missing observations for any of the variables selected for analysis. The child- and caregiver-level characteristics of study participants are shown in Table 1.

**Table 1:** Characteristics of participants and prevalence of receipt of SMC by ineligible children

Variables	Total (N = 3299)		Received SMC		p-value
	Number	%	Number	%	
All children	3299	100	1000	30.3	
Child age					
5	365	11.1	306	83.8	
6	915	27.7	300	32.8	
7	779	23.6	195	25	
8	584	17.7	96	16.4	< 0.001**
9	456	13.8	62	13.6	
10	200	6.1	41	20.5	
Child sex					
Female	1627	49.3	480	29.5	0.347
Male	1672	50.7	520	31.1	
Caregiver age					
Less 20 years	110	3.3	41	37.3	0.015**
20–39 years	2673	81	778	29.1	
40 years and above	516	15.6	181	35.1	
Caregiver gender					
Female	2944	89.2	861	29.2	
Male	355	10.8	139	39.2	0.001**
Caregiver literacy					
No	1186	36	367	30.9	
Yes	2113	64	633	30	0.652
Caregiver level of education					
None	725	22	208	28.7	
Informal or religious education	838	25.4	305	36.4	
Primary school	528	16	151	28.6	
Secondary school	881	26.7	243	27.6	0.001**
Higher education	327	9.9	93	28.4	
Caregiver marital status					
Not married	195	5.9	62	31.8	0.680
Married	3104	94.1	938	30.2	
Caregiver occupation					
Unemployed	1065	32.3	316	29.7	0.670
Employed	2234	67.7	684	30.6	
Caregiver knowledge of purpose of SMC					
No	232	8.6	64	27.6	0.561
Yes	2463	91.4	736	29.9	
Caregiver confidence (belief) that SMC is effective in preventing malaria					
No	104	3.9	16	15.4	
Yes	2591	96.1	784	30.3	0.005**
Community distributor observed DOT					
No	401	13.1	113	28.2	
Yes	2657	86.9	856	32.2	0.156
Caregiver knew SMC age eligibility					
No	268	9.9	94	35.1	
Yes	2427	90.1	706	29.1	0.049

** p ≤ 0.05

### Prevalence of receipt of SMC medicines by ineligible children aged 5–10 years

Table 1 presents the prevalence of SMC receipt among age-ineligible children by various child, caregiver and household variables. Among the 3299 age-ineligible children analysed, 1000 (30.3%) received SMC medicines. The majority (60.6%) of children who received SMC were 5- and 6-year-olds. Receipt of SMC medicines was highest among children aged 5 years (83.8%), with the proportion of SMC recipients generally decreasing as children aged older.

### Factors associated with the receipt of SMC medicines by age-ineligible children

**Fig. 2:**
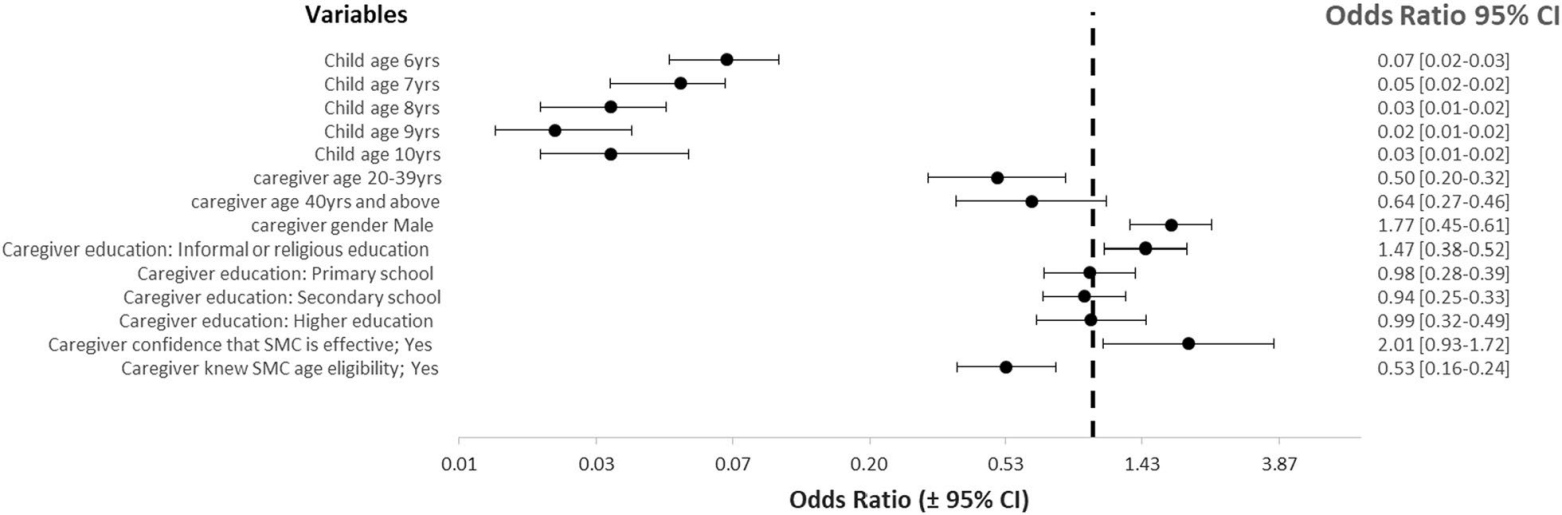
Forest plot of adjusted odds ratios of factors considered in the multivariable regression model

Table 2 and Fig. 2 present results of adjusted odds ratios across various child- and caregiver-level factors included in the mixed-effects multivariable logistic regression model. Compared with 5 year-olds, children who were older than 5 years had significantly lower odds of receiving SMC medicines (6 year-olds: OR: 0.07, 95% CI 0.05–0.10, p < 0.001; age 7 year-olds: OR: 0.05, 95% CI 0.03–0.07, p < 0.001; age 8 year-olds: OR: 0.03, 95% CI 0.02–0.05, p < 0.001; 9 year-olds: OR: 0.02, 95% CI: 0.01—0.04, p < 0.001; 10 year-olds: OR: 0.03, 95% CI 0.02–0.05, p < 0.001). Some caregiver-level characteristics were found to be significantly associated with the administration of SMC medicines to children who were age-ineligible for SMC. Age ineligible children of caregivers aged 20–39 years were less likely to receive SMC compared with those of younger caregivers aged less than 20 years (OR: 0.5, 95% CI 0.30–0.82, p = 0.006). Higher odds of receiving SMC medicines were found among children whose primary caregivers were male at the time of survey relative to those whose caregivers were female (OR: 1.77, 95% CI 1.32–2.38, p < 0.001). Similarly, age-ineligible children of caregivers with informal or religious education were more likely to receive SMC medicines compared with those whose children had no education or other forms of education.

**Table 2:** Results of multivariate logistic regression model of receipt of seasonal malaria chemoprevention by age-ineligible children and associated factors in nine States in Nigeria (n = 3299)

Variables	Crude measures of association		Adjusted measures of association	
	Crude odds ratio (95% CI)	p value	Adjusted odds ratio (95% CI)	p value
Child age				
5	Reference		Reference	
6	0.06 (0.04–0.086)	< 0.001**	0.07 (0.046–0.102)	< 0.001**
7	0.04 (0.026–0.057)	< 0.001**	0.05 (0.03–0.069)	< 0.001**
8	0.02 (0.015–0.036)	< 0.001**	0.03 (0.018–0.045)	< 0.001**
9	0.02 (0.011–0.028)	< 0.001**	0.02 (0.013–0.035)	< 0.001**
10	0.03 (0.016–0.046)	< 0.001**	0.03 (0.018–0.053)	< 0.001**
Caregiver age				
less 20 years	Reference		Reference	
20–39 years	0.52 (0.322–0.85)	0.009**	0.50 (0.302–0.821)	0.006**
40 years and above	0.71 (0.418–1.199)	0.199	0.64 (0.371–1.103)	0.108
Caregiver gender				
Female	Reference		Reference	
Male	1.83 (1.374–2.436)	0.001**	1.77 (1.317–2.378)	0.001**
Caregiver level of education				
None	Reference		Reference	
Informal or religious education	1.60 (1.218–2.114)	0.001**	1.47 (1.087–1.991)	0.020**
Primary school	1.05 (0.764–1.452)	0.751	0.98 (0.703–1.365)	0.012**
Secondary school	1.01 (0.758–1.338)	0.961	0.94 (0.695–1.271)	0.902
Higher education	1.11 (0.761–1.624)	0.585	0.99 (0.667–1.48)	0.687
Caregiver confidence (belief) that SMC is effective in preventing malaria				
No	Reference		Reference	
Yes	1.88 (1.031–3.426)	0.039**	2.01 (1.07–3.723)	0.030**
Caregiver knowledge of SMC age eligibility				
No	Reference		Reference	
Yes	0.52 (0.365–0.74)	< 0.001**	0.53 (0.372–0.765)	< 0.001**

** p ≤ 0.05

Some SMC-specific variables were found to be significantly associated with the administration of SMC medicines to children who were age-ineligible for SMC. Lower odds of age-ineligible children receiving SMC medicines were observed among caregivers who were knowledgeable of SMC age eligibility (OR: 0.53, 95% CI 0.37–0.77, p < 0.001), compared with those who were not knowledgeable of age eligibility. Conversely, higher odds of receipt of SMC medicines were found among age-ineligible children whose caregivers were more confident that SMC is effective in preventing malaria (OR: 2.01, 95% CI 1.07–3.72, p = 0.030), compared with those whose caregivers were less confident in SMC’s effectiveness in preventing malaria.

## Discussion

In addition to estimating the magnitude of receipt of SMC medicines by ineligible older children, the results show various child, caregiver and SMC-related factors associated with the issue using data collected during comprehensive end-of-round surveys in nine Nigerian states in 2022. The study found that about a third of age-ineligible older children sampled received SMC medicines during the 2022 SMC campaigns. This finding lends support to available, though limited, evidence that the administration of SMC medicines to ineligible children is a common occurrence [14]. Administration of SMC medicines to children who do not meet the eligibility criteria for SMC based on their age has various implications and presents numerous challenges as outlined in the following sections.

As the current formulations and dosing of the medicines used in SMC campaigns in Nigeria are intended for children younger than 5 years, it is possible that administering those medicines to older children represents underdosing. Exposure of children to sub-optimal doses can contribute to the development of parasite resistance [4, 15]. If left unaddressed, this is likely to undermine the programmatic effectiveness of SMC and may weaken the level of confidence that communities have in SMC as a malaria preventive intervention. It was found that the majority of ineligible children who received SMC were aged 5 to 6 years. As with other medicines, pharmacometrics of SMC medicines depend primarily on children’s weight [19]. Hence, administration of SMC medicines to children that are just above the eligible age range, whose weight is therefore unlikely to be substantially higher, is less likely to constitute underdosing. Based on the study findings and considering uncertainties about the exact extent to which SMC may be contributing to the development of resistance in settings in the Sahel region of West and Central Africa [6], the observed trend of receipt of SMC predominantly by children aged 5 to 6 years may be less likely to pose a significant risk of development of drug resistance. The finding that about 40% of older children who received SMC medicines were much older than 5 years is, nonetheless, concerning giving the implications of sub-optimal dosing in those children. It is an intriguing finding that such a large proportion of much older children received SMC medicines despite the obvious age difference between them and under-5 children. However, such children may not be distinguishable from under-5 children by mere physical appearance, due to the prevalence of malnutrition and stunting in the study setting, and SMC community distributors’ difficulty in ascertaining children’s age due to absence of verifiable home-based birth and civil registration records [8–11]. From an operational perspective, however, administering SMC medicines to ineligible children presents a substantial challenge, particularly in terms of ensuring the availability of sufficient SPAQ stock levels to reach the entire target population of eligible children. This has important implications for reaching and sustaining high target population coverage, while maintaining optimal levels of programmatic impact and effectiveness in SMC delivery settings. From an economic perspective, it is likely that administration of SMC medicines to ineligible children increases the cost of SMC and reduces its cost-effectiveness. This is because cost-effectiveness decreases if medicines are given to individuals who are unlikely to benefit, which may also result in a decrease of coverage among eligible children.

The study has identified various child, caregiver and SMC-related factors that influence the receipt of SMC medicines by ineligible populations of older children. It found that children’s age, and caregivers’ characteristics like age, gender, level of education and employment status were significantly associated with the issue. These findings are consistent with previous evidence that caregiver gender, age and other sociodemographic characteristics can influence children’s uptake of preventive health services and health outcomes [19, 20]. The findings thus underscore the need for context-specific community engagement efforts in future SMC campaigns to be tailored to address these predisposing factors, such as by strengthening knowledge of SMC age eligibility and its importance among younger and male caregivers, and those in households with older children in the 5- to 6-year age range. These can be achieved by reinforcing the training of SMC community distributors and community engagement personnel; equipping them with the skills required for effective communication of information on SMC eligibility criteria, their importance and other SMC-related information; and improving their competence in tailoring communication strategies to specific household and community level contexts during SMC campaigns.

The study found considerable relationships between SMC-specific variables and the receipt of SMC medicines by ineligible children. It is important to note in addition to challenges faced by community distributors in determining children’s exact age, administration of SMC medicines to ineligible children may also reflect the pressure from caregivers on community distributors to provide SMC medicines to older children, as supported by the finding of higher odds of receipt of SMC medicines among age-ineligible children whose caregivers were more confident in SMC’s effectiveness as a malaria prevention intervention. Additionally, it is likely that doses of SMC medicines left with caregivers by community distributors to administer to age eligible children on the subsequent 2 days following the first dose, were administered to older siblings and other older children in the household for the same reason. As acknowledged earlier, these present a substantial challenge for ensuring that the target population of eligible children are reached and that they receive the complete number of doses of SMC medicines.

To address these challenges, it is pertinent to improve the competence of community distributors in determining children’s age with available home-based records, such as by requesting birth certificates, immunization cards and medical prescriptions for a previous illness. Also, SMC personnel should be better trained on strict compliance with SMC eligibility criteria, even when under pressure to provide SMC medicines to ineligible children. It is also important that community distributors are able to determine children’s age in the absence of verifiable records. One strategy that has been adopted in some of implementation settings is the use of historical prompts to validate caregivers’ reports of children’s age. Findings also underscore the need for future SMC campaigns to improve and address gaps in caregivers’ knowledge of SMC age eligibility as illustrated by the findings of higher odds of age-ineligible children receiving SMC medicines among caregivers who were less knowledgeable of SMC age eligibility. This can be achieved through pre-cycle SMC awareness and sensitization campaigns, and by leveraging interactions of community distributors with caregivers to boost their confidence in the effectiveness of SMC while also strengthening their knowledge and perceptions regarding age eligibility and its importance.

### Strengths and limitations

The study is one of the earliest attempts to quantity the extent to which ineligible children are receiving SMC medicines. The large dataset used, and statistical power of the analyses are major merits of the study. The mixed-effects regression approach use in fitting the model, with random intercepts for cluster units (wards) has the advantage of being to appropriately model data with observations that are nested in hierarchical data as with the dataset used in the study, while helping to account for the potential for clustering effect [21]. Overall, the findings provide important evidence on the magnitude and factors associated with the problem, thereby providing useful insights that can help to guide the articulation and deployment of context-specific programme improvement strategies in future SMC campaigns.

A major limitation of the study is that the analytic sample may not be representative of the general population of children aged 5–10 years in the study setting, as ineligible children were sampled from households with SMC eligible children during the survey. It is likely that the current estimates of receipt of SMC by ineligible children are overestimated given this consideration. As such, the findings may not have limited generalizability to the wider population of older children in the study setting or similar contexts. Another limitation is the survey’s reliance on self-reporting of children’s age by caregivers which could have resulted in misclassification of children’s SMC age-eligibility status. There was also reliance on caregivers’ recall of children’s receipt of SMC medicines, which may be prone to social desirability and recall biases.

### Implications for further research

Given these limitations, future assessments could consider a more representative sample of older children by sampling from households irrespective of whether they have SMC-eligible children or not. Further quantitative studies may need to assess factors influencing administration of SMC to age-ineligible children from the perspectives of other stakeholders such as community distributors. The findings also underscore the need for qualitative studies to explore and more deeply understand the individual and contextual factors associated with the administration of SMC medicines to children who are ineligible to receive the medicines. Furthermore, future research may also be needed to assess the impact of such occurrences on SMC coverage, adherence to the full 3-day course, effectiveness, cost-effectiveness, and the prevalence of resistance markers.

## Conclusions

This study contributes important evidence on the magnitude of the receipt of SMC by age-ineligible children, while identifying individual and contextual factors associated with it. Significant associations were found between receipt of SMC medicines by ineligible children and child-level factors such as age, as well as caregiver-level factors such as age, gender, knowledge of SMC age eligibility and confidence (belief) in the protective effect of SMC. The findings provide potentially useful insights that can help inform and guide context-specific SMC implementation quality improvement efforts.

## Data Availability

Data employed in this study are available from the authors upon reasonable request.
