# Correction: Receipt of seasonal malaria chemoprevention by age-ineligible children and associated factors in nine implementation states in Nigeria

**DOI:** 10.1186/s12936-024-04942-x

**Published:** 2024-04-23

**Authors:** Taiwo Ibinaiye, Kunle Rotimi, Ayodeji Balogun, Adaeze Aidenagbon, Chibuzo Oguoma, Christian Rassi, Kevin Baker, Olusola Oresanya, Chuks Nnaji

**Affiliations:** 1 Malaria Consortium, 33 Pope John Paul Street, Maitama, Abuja, FCT Nigeria; 2 Malaria Consortium, No. 24 Randa Area, Ogbomoso, Nigeria; 3 Malaria Consortium, The Green House, 244-254 Cambridge Heath Road, London, E2 9DA UK; 4 Department of Global Public Health, Karolinska Institute, Stockholm, Sweden

**Correction: Malaria Journal (2024) 23:91** 10.1186/s12936-024-04916-z

Following publication of the original article [1], it came to the authors' attention that there was an error in the Abstract:

where it says “There were lower odds of an age-ineligible child receiving SMC medicines among caregivers who were knowledgeable of SMC age eligibility, compared with those who were not knowledgeable of age eligibility.”

it said “There were lower odds of an age-ineligible child receiving SMC among caregivers who had knowledge of SMC age eligibility, compared with those who were knowledgeable of age eligibility”.

The article has since been corrected. The authors thank you for reading this erratum and apologize for any problems caused.
